# Analyzing road traffic crashes through multidisciplinary video data approaches

**DOI:** 10.3389/fpubh.2025.1614017

**Published:** 2025-12-11

**Authors:** Haiqing Zhang, Yongqiang Shang

**Affiliations:** 1 College of Information Engineering, Shaanxi A&F Technology University, Yangling, Shaanxi, China; 2 School of Management, Wuhan University of Technology School of Management, Wuhan, Hubei, China; 3 Department of Information Engineering, Information Engineering Department, Xinyang Agriculture and Forestry University, Xinyang, Hennan, China

**Keywords:** traffic safety, risk modeling, neural relational networks, video analytics, multi-agent systems

## Abstract

**Introduction:**

The rising incidence of road traffic crashes has emerged as a pressing global concern, representing a multifaceted challenge to public health, urban safety, and sustainable mobility. With rapid urbanization and increasing vehicular density, traffic environments have become highly dynamic, multi-agent systems characterized by complex interactions among heterogeneous road users, including vehicles, cyclists, and pedestrians. These interactions occur within high-dimensional and context-rich environments that are difficult to model using traditional approaches. Consequently, there is an urgent need for robust, data-driven frameworks capable of capturing the intricacies of real-world traffic scenarios.

**Methods:**

In this study, we propose a novel computational framework designed to enhance the analysis of traffic safety through a multidisciplinary lens. Our approach integrates advanced video data analytics with artificial intelligence techniques, emphasizing the fusion of spatiotemporal modeling, behavioral analysis, and environmental context.

**Results:**

By leveraging large-scale, *in-situ* video data from urban intersections and road networks, our method provides a granular understanding of risk factors and interaction patterns that precede collisions or near-miss events.

**Discussion:**

This framework is tailored for the challenges posed by complex, heterogeneous traffic systems and aligns with current research interests in deploying AI for risk-sensitive, behavior-aware decision-making within urban mobility infrastructures.

## 1 Introduction

In recent years, the escalating incidence of road traffic crashes has emerged as a critical public safety concern worldwide, prompting the urgent need for more effective analysis methodologies (1). Traditional data sources such as police reports or crash statistics offer limited insights into the underlying causes of traffic incidents (2). Not only do these methods often lack contextual richness, but they also struggle to account for behavioral, environmental, and infrastructural dynamics. With the proliferation of video surveillance infrastructure and advancements in computer vision, video data has surfaced as a valuable yet underutilized resource in traffic crash analysis (3). This video data not only enables detailed post-crash analysis through frame-by-frame review but also allows for real-time monitoring and proactive safety interventions (4). Moreover, when integrated with multidisciplinary approaches that include behavioral science, traffic engineering, and data analytics, video-based methods can offer comprehensive insights that were previously unattainable (5). Thus, analyzing road traffic crashes through multidisciplinary video data approaches represents a necessary evolution in traffic safety research—enhancing our ability to understand, prevent, and mitigate the impact of such incidents (6).

Early efforts to understand crash events using video data concentrated on manually designed systems that emulated human reasoning through structured logic and predefined analytical frameworks (7). These systems typically interpreted crash footage by mapping observed traffic events to known risk conditions, using deterministic rule sets based on expert recommendations (8). For instance, analysts would encode if-then rules regarding driver behavior or intersection violations, allowing the system to flag hazardous scenarios like red-light running or sudden lane shifts (9). While this method allowed for explainable decision-making and incorporated domain-specific expertise, it struggled with scalability and was sensitive to ambiguities common in real-world video, such as partial occlusions, low-resolution footage, or unpredictable behaviors (10). Moreover, the development and maintenance of such rule-based systems were labor-intensive, limiting their applicability for large-scale or real-time analysis (11).

As video data became more abundant and diverse, researchers began utilizing pattern-recognition techniques that could infer meaningful structures from annotated examples rather than relying on predefined instructions (12). These systems were trained on curated crash datasets to identify potentially dangerous behaviors, classify crash types, or detect near-miss events by analyzing spatiotemporal patterns in video footage (13). Machine learning algorithms such as support vector machines and random forests were applied to extract relevant features like vehicle trajectories, speed profiles, and collision risk zones from sequential frames (14, 15). Although these approaches improved performance by incorporating temporal context and reducing reliance on hardcoded rules, they still faced constraints in generalizing across varied environments due to differences in lighting, weather, camera perspectives, and road layouts. In addition, the success of these models often hinged on manual feature selection, which introduced biases and limited the discovery of complex patterns within raw video streams (16).

To overcome the limitations of handcrafted features and enhance the capacity for generalization, researchers have increasingly adopted deep learning models capable of end-to-end video understanding (17). These models leverage layered neural architectures such as convolutional and recurrent networks to extract spatial and temporal dependencies directly from pixel-level inputs (18). State-of-the-art systems, including I3D, SlowFast, and Transformer-based video models, are pre-trained on large-scale video corpora and then fine-tuned for crash detection tasks, enabling accurate localization, classification, and behavioral inference from video feeds (19). Furthermore, multimodal learning strategies have enabled these systems to integrate visual data with auxiliary information—such as traffic sensor outputs, weather conditions, or real-time vehicle metadata—providing a richer analytical framework for understanding crash causality (20). Despite their superior capabilities, these models often operate as opaque decision engines, making them difficult to interpret and verify in high-stakes scenarios. Nevertheless, the convergence of deep learning and multidisciplinary traffic analysis continues to redefine the landscape of road safety research and offers promising solutions for proactive crash prevention and response (21).

To overcome the interpretability issues of deep learning models and the scalability limitations of symbolic and data-driven approaches, our method proposes a hybrid framework that leverages spatiotemporal deep learning enhanced by interpretable causal inference modules. This approach is informed by the need to bridge the gap between accuracy and explainability in traffic crash analysis. Our method integrates video-based object tracking and behavior prediction with a knowledge-guided reasoning layer that can infer causality from observed patterns. By combining data-driven insights with structured reasoning, we aim to develop a system that is both accurate in detection and transparent in its diagnostic capability. Furthermore, to ensure adaptability across diverse urban and rural traffic contexts, we incorporate a domain generalization module that calibrates model outputs based on environmental features and video characteristics. This addresses the variability challenges encountered in earlier machine learning models. Ultimately, our approach embodies a multidisciplinary fusion that unifies visual computing, AI reasoning, and traffic safety theory, setting the stage for robust and interpretable crash analysis.

This study contributes to the field in the following ways. First, we propose a hybrid video-based analytical framework that fuses deep spatiotemporal modeling with interpretable causal reasoning to analyze road traffic crashes. Second, we develop a relational graph-based encoder to capture heterogeneous agent interactions and dynamic risk patterns. Third, our hierarchical disentanglement strategy separates local motion anomalies from semantic inconsistencies, enabling interpretable scene-level risk assessment. Lastly, we conduct extensive evaluations on multiple real-world traffic datasets, demonstrating state-of-the-art performance and robustness. These contributions directly address the limitations of prior work in scalability, explainability, and generalization. To ground our proposed framework within the existing body of knowledge, the next section surveys recent developments in multimodal traffic surveillance, behavioral modeling, and crash reconstruction.

## 2 Related work

### 2.1 Multimodal traffic surveillance analysis

The integration of multimodal data sources in traffic surveillance has significantly advanced the understanding of road traffic crashes. Traditional single-sensor systems often fail to capture the complexity and causality of crash events (22). In contrast, modern approaches combine video feeds, vehicle telemetry, environmental sensors, and geographic data to enable a holistic reconstruction and analysis of traffic incidents (23). High-resolution cameras, coupled with computer vision algorithms, can detect and track vehicles and pedestrians, offering temporal and spatial context to crash occurrences. These visual inputs are often synchronized with accelerometer data, GPS logs, and weather sensors to provide multidimensional insights into crash dynamics (24). Deep learning architectures, such as convolutional neural networks (CNNs) and recurrent neural networks (RNNs), are widely employed for object detection, motion prediction, and behavior analysis from video data (25). The fusion of video with other modalities enhances the model's ability to infer pre-ash conditions, such as abrupt lane changes, braking patterns, or driver distraction (26). Researchers have developed frameworks that apply multimodal embeddings to correlate driver actions with external stimuli, aiding in root cause analysis of crashes. The synchronization and alignment of data across modalities remain challenging, but progress in sensor calibration and temporal data alignment techniques has improved the accuracy and reliability of crash reconstructions (27). Applications of these approaches extend beyond post-crash forensics to real-time crash prediction and prevention. Systems are being deployed to flag anomalous driving behavior or environmental conditions that typically precede accidents. This line of research bridges engineering, computer science, and cognitive psychology, illustrating the necessity of multidisciplinary collaboration in traffic safety analysis (28).

### 2.2 Human behavior and cognitive modeling

Understanding human behavior in traffic environments is crucial for analyzing crash causation and developing preventive strategies (29). Video-based behavior modeling allows researchers to study not only vehicle trajectories but also driver and pedestrian decision-making processes. Eye-tracking data, head posture estimation, and facial expression analysis from in-vehicle cameras have been instrumental in assessing driver attentiveness, cognitive load, and emotional states (30). Similarly, pedestrian behavior near crosswalks or intersections can be modeled to predict risk-prone actions based on body posture, gaze direction, and hesitation patterns. Behavioral models are often grounded in psychological and cognitive theories, such as the Theory of Planned Behavior and Situation Awareness models. These frameworks are operationalized using machine learning techniques to predict human responses to dynamic traffic stimuli. For instance, probabilistic graphical models and deep reinforcement learning are used to simulate decision-making under uncertainty, capturing phenomena such as delayed reaction times or risk compensation behaviors (31). The availability of large-scale naturalistic driving datasets with synchronized video and behavioral annotations has enabled the training of robust models that generalize across diverse environments and driver demographics. However, individual variability in behavior remains a significant challenge, prompting research into personalized modeling that accounts for driver-specific risk profiles and historical behavior (32). Moreover, cross-disciplinary efforts have led to the development of explainable AI systems that can interpret driver actions in terms of cognitive and situational factors, enhancing the interpretability of automated crash analysis tools (33). This convergence of behavioral science and computational modeling is instrumental in designing interventions, such as driver monitoring systems and adaptive human-machine interfaces, aimed at reducing crash likelihood (34).

### 2.3 Crash scene reconstruction and simulation

Crash scene reconstruction using video data and simulation techniques has emerged as a powerful tool for understanding the mechanics and dynamics of road traffic crashes (35). This direction leverages computer vision, physics-based modeling, and forensic analysis to recreate the sequence of events leading to a collision. Keyframe extraction, trajectory estimation, and 3D scene reconstruction enable precise visualization of crash scenarios. These reconstructions serve as inputs for simulation platforms that validate hypotheses about causality and contributory factors (36). Simulation-based approaches utilize software such as PC-Crash, VISSIM, and SUMO, often enhanced with real-world video data to calibrate models. These platforms simulate vehicle kinematics, road geometry, and driver inputs under various conditions to reproduce crash scenarios with high fidelity. When combined with high-definition maps and environmental data, such simulations can explore counterfactual scenarios—what-if analyses—to evaluate the impact of alternative road designs, vehicle technologies, or traffic regulations (37). Machine learning algorithms contribute to the automation of video-based reconstruction by detecting collision points, estimating vehicle speeds, and identifying object deformation patterns. Techniques such as optical flow analysis and pose estimation are critical in deriving motion dynamics from 2D videos, which are then extrapolated into 3D simulations (38). This research direction is particularly relevant to policy-making and litigation, as it provides objective, data-driven insights into crash causality. It also supports the development of virtual testing environments for autonomous vehicle safety and the assessment of crash avoidance systems. Interdisciplinary collaboration with transportation engineers, legal experts, and law enforcement ensures that the reconstructed scenarios are not only technically accurate but also socially and legally interpretable (39).

The following sections detail the experimental setup and results, demonstrating how each component of the proposed methodology contributes to improved risk detection and interpretability in real-world traffic scenarios.

## 3 Method

### 3.1 Overview

Traffic safety remains a pivotal research area due to its direct impact on public health, urban development, and transportation systems. The complexity of traffic environments, characterized by heterogeneous participants such as vehicles, pedestrians, and cyclists, presents significant challenges for designing effective safety mechanisms. Consequently, the field has witnessed a surge of interdisciplinary efforts aiming to model, predict, and mitigate safety risks in traffic systems. This section introduces the methodology we develop to address traffic safety from a computational and algorithmic standpoint. We structure the method into three core components, each corresponding to a subsequent subsection. Each component tackles a crucial aspect of the traffic safety modeling pipeline, ranging from formal problem definition to model construction and strategic reasoning under uncertainty. In Section 3.2, we provide a formalized representation of traffic safety risk modeling. This includes defining agent interactions in traffic scenarios, encoding the spatial-temporal behavior of multiple agents, and identifying critical variables that influence collision likelihoods. We introduce notations to mathematically represent the evolution of trajectories and contextual dependencies, while outlining the general objective of our approach. Section 3.3 presents our core model, termed Neural Relational Trajectory Encoder (NRTE), which is designed to capture relational dependencies between heterogeneous traffic participants. This model employs a graph-based recurrent neural structure that enables information propagation through dynamically constructed interaction graphs. Unlike traditional models that assume fixed spatial neighborhoods or handcrafted interaction rules, NRTE adapts relational encoding based on context-aware attention mechanisms, allowing for flexible reasoning over complex scenarios, including occlusions, trajectory overlaps, and non-linear accelerations. Section 3.4 describes our strategic framework named Hierarchical Scene Risk Disentanglement (HSRD). This strategy introduces a multi-scale reasoning scheme to distinguish between low-level motion anomalies and high-level semantic threats. The disentanglement is achieved through a dual-channel inference process including one capturing localized risk cues and the other learning semantic abstractions of scene-level hazards. This dualism enables interpretable and modular analysis of safety-critical events and facilitates downstream tasks such as traffic incident forecasting and preventative policy learning.

Our proposed methodology is rooted in the hypothesis that traffic risk emerges not only from isolated agent behaviors but also from their latent relational configurations and contextual semantics. To this end, the integration of spatiotemporal structure, neural relational modeling, and hierarchical risk inference forms the backbone of our traffic safety framework. This unified approach is designed to address the non-stationarity, heterogeneity, and partial observability intrinsic to real-world traffic data. Through the subsequent sections, we elaborate on each of these methodological pillars with rigorous formalism and design intuition, paving the path toward a robust and interpretable model of traffic safety.

### 3.2 Preliminaries

Let S denote the space-time scene of a traffic environment, composed of dynamic agents such as vehicles, pedestrians, and cyclists interacting within a geographical region over time. Each agent $a_{i}\in A$ follows a trajectory $\tau_{i}={\left\{x_{i}^{t}\right\}}_{t=1}^{T}$, where $x_{i}^{t}\in {\mathbb{R}}^{d}$ represents its state at time *t*, including position, velocity, heading, and potentially higher-order dynamics.

The scene at any time *t* is represented as a configuration $C^{t}=\left\{x_{1}^{t},x_{2}^{t},\ldots ,x_{N}^{t}\right\}$, where N=|A| is the number of agents. The goal of traffic safety modeling is to estimate a risk score $R^{t}\in \mathbb{R}$ associated with $C^{t}$, capturing the likelihood of unsafe events within a short future horizon Δ*t*.

To formalize this, we define a risk function R as a mapping over a temporal window of *k* past frames.


$$\begin{array}{l} R^{t}=f\left(C^{t-k:t}\right) \end{array}$$ (1)

Each agent's trajectory is governed by a latent intent $z_{i}\in Z$ and control policy π_*i*_, influenced by internal dynamics and exogenous control inputs. The effect of interactions is captured via a time-indexed graph $G^{t}=\left(A,E^{t}\right)$, with edges $e_{ij}^{t}$ encoding relational context. A relational encoder lifts raw trajectories and interactions into a high-dimensional spatiotemporal feature space $H^{1:T}$ that captures motion evolution and agent dependencies.

To quantify risk, we introduce a scene-level hazard scoring function that aggregates relational threats between agents.


$$\begin{array}{l} R^{t}=\underset{\left(i,j\right)\in E^{t}}{\sum }\rho \left(x_{i}^{t},x_{j}^{t},\Delta x_{ij}^{t}\right) \end{array}$$ (2)

We enrich the interaction representation with critical indicators such as time-to-collision (TTC) and minimal distance violation (MDV), which provide interpretable proxies for collision imminence and spatial encroachment, respectively. These are fused into edge features for dynamic risk evaluation.


$$\begin{array}{l} e_{ij}^{t}\leftarrow \phi \left(x_{i}^{t},x_{j}^{t},{\text{TTC}}_{ij}^{t},{\text{MDV}}_{ij}^{t}\right) \end{array}$$ (3)

Under partial observability, each observed state $x_{i}^{t}$ is assumed to be influenced by an unobserved latent variable $h_{i}^{t}$, which accounts for sensing noise or behavioral uncertainty. We propagate beliefs over time via recursive Bayesian updates, enabling temporal filtering of uncertain states. Aggregating risk across agents and time yields a probabilistic traffic risk estimator.


$$\begin{array}{l} \mathbb{P}\left(\text{Unsafe Event}|C^{t-k:t}\right)=\sigma \left(\underset{i,j}{\sum }w_{ij}^{t}\cdot \rho \left(e_{ij}^{t}\right)\right) \end{array}$$ (4)

In regard to the recursive Bayesian update described in Equation (4), this mechanism addresses partial observability by estimating latent variables that represent uncertain or noisy agent states. In real-world video surveillance scenarios, visual degradation due to occlusion, lighting variation, or motion blur can result in incomplete or corrupted state observations. The latent variable $h_{i}^{t}$ functions as a probabilistic encoding of the true underlying state, updated recursively as new observations become available. Through Bayesian filtering, the model refines its belief over time, adjusting risk estimations in the presence of fluctuating observation quality. This formulation allows the risk predictor to integrate uncertainty into its reasoning process, producing probabilistic risk estimates that are robust to real-world sensing noise and missing data. As a result, the proposed framework remains effective even under imperfect input conditions and supports more reliable decision-making in dynamic traffic environments.

To ensure scalability, the edge set is pruned through relevance criteria, typically involving spatial proximity and TTC thresholds, reducing computational burden while preserving salient risks. The overall safety assessment objective integrates these local risk measures into a global inference target.


$$\begin{array}{l} J_{\text{safety}}=\int_{S}\mathbb{P}\left(\text{Unsafe Event}|C\right)\cdot 𝕀\left[\text{event in horizon}\right]dC \end{array}$$ (5)

### 3.3 NRTE

**Figure 1:**
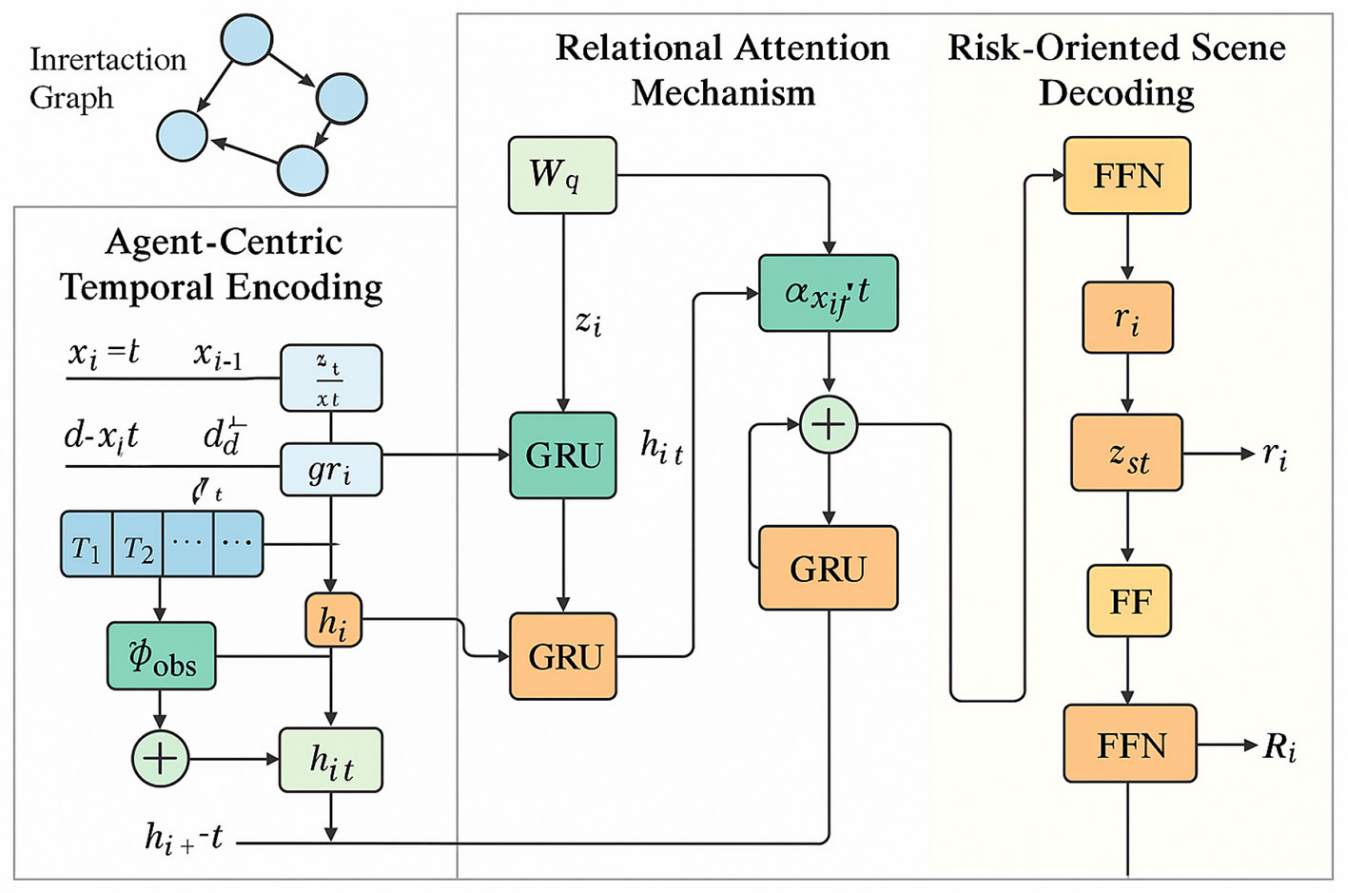
An illustration of the NRTE. The NRTE framework illustrates the three main components: Agent-Centric Temporal Encoding, which models individual agent trajectories with temporal dynamics, latent behavioral codes, and motion derivatives; Relational Attention Mechanism, which captures inter-agent interactions through attention-weighted message passing on a dynamic interaction graph; and Risk-Oriented Scene Decoding, which integrates agent-level and scene-level representations to estimate probabilistic risk for both individuals and the overall traffic scene.

To effectively capture the spatiotemporal dependencies and latent interaction structures within complex traffic scenes, we propose a novel model named Neural Relational Trajectory Encoder (NRTE). This model operates on dynamic interaction graphs and encodes multi-agent trajectories by leveraging both relational inductive bias and neural sequence modeling (as shown in Figure 1).

#### 3.3.1 Agent-centric temporal encoding

The fundamental concept of NRTE lies in establishing a temporally dynamic interaction graph $G^{t}=\left(A,E^{t}\right)$, where A denotes the set of traffic agents and $E^{t}$ represents the directed relations among them at time step *t*. This graph-based formulation allows for an explicit representation of both the time-dependent behavior of individual agents and their interdependent relationships. Each agent node is equipped with a history of trajectory states, which are encoded into latent dynamics-aware features through a recurrent architecture. These features capture not only the agent's motion but also its behavioral evolution.

Let the raw trajectory of agent *a*_*i*_ over *T* time steps be represented as ${\text{x}}_{i}^{1:T}={\left\{x_{i}^{t}\right\}}_{t=1}^{T}$, where $x_{i}^{t}\in {\mathbb{R}}^{d}$ denotes its spatial and kinematic state at time *t*. To process this temporal sequence, we employ a gated recurrent unit (GRU) to sequentially encode observations into hidden representations.


$$\begin{array}{l} {\text{h}}_{i}^{t}=\text{GRU}\left({\text{h}}_{i}^{t-1},\phi_{\text{obs}}\left(x_{i}^{t}\right)\right) \end{array}$$ (6)

Here, $\phi_{\text{obs}}:{\mathbb{R}}^{d}\to {\mathbb{R}}^{h}$ is a neural observation encoder that projects each raw state into an embedding space ℝ^*h*^ to account for non-linear and multimodal features present in traffic behavior.

To enhance the temporal expressiveness of ${\text{h}}_{i}^{t}$, we incorporate residual temporal aggregation across previous steps, allowing the model to retain long-range dependencies. This mechanism is expressed.


$$\begin{array}{l} {\tilde{\text{h}}}_{i}^{t}={\text{h}}_{i}^{t}+\overset{k}{\underset{\tau =1}{\sum }}\gamma^{\tau }\cdot {\text{h}}_{i}^{t-\tau } \end{array}$$ (7)

The decay coefficient γ∈(0, 1) governs the contribution of prior memory states, while *k* is a configurable memory horizon.

Further, to distinguish between static behavioral patterns and time-varying intentions, we enrich the GRU input with latent behavior priors. Let $z_{i}^{t}$ denote an internal latent code drawn from a prior behavior distribution conditioned on past motion.


$$\begin{array}{l} z_{i}^{t}\sim p\left(z_{i}^{t}|{\text{x}}_{i}^{1:t-1}\right),\;{\hat{x}}_{i}^{t}=\left[x_{i}^{t};z_{i}^{t}\right] \end{array}$$ (8)

The concatenated vector ${\hat{x}}_{i}^{t}$ then replaces $x_{i}^{t}$ as input to the observation encoder, enabling temporal representations to reflect intention-aware motion dynamics.

The model also considers local motion curvature and velocity changes as first-order derivatives.


$$\begin{array}{l} {ẋ}_{i}^{t}=x_{i}^{t}-x_{i}^{t-1},\;{ẍ}_{i}^{t}={ẋ}_{i}^{t}-{ẋ}_{i}^{t-1} \end{array}$$ (9)

These derivatives are fused with positional input to provide rich temporal motion encoding that captures acceleration, turning patterns, and abrupt maneuvers critical to risk-sensitive modeling.

To preserve geometric invariance and model local positional patterns, we embed relative displacements between consecutive frames.


$$\begin{array}{l} \delta x_{i}^{t}=\phi_{\text{disp}}\left(x_{i}^{t}-x_{i}^{t-1}\right) \end{array}$$ (10)

where ϕ_disp_ is a shared feed-forward network capturing displacement encoding invariant to global coordinates.

All gated recurrent units (GRUs) within the agent-centric temporal encoder are initialized using Xavier uniform initialization for weight matrices and zero initialization for bias terms, ensuring stable gradient propagation during training. The latent behavioral code $z_{i}^{t}$ is sampled from a standard multivariate Gaussian distribution N(0,I) during training. Feature projection modules, including ϕ_*obs*_, ϕ_*disp*_, and ϕ_*rel*_, are implemented as two-layer feedforward neural networks with ReLU activations, and initialized via Xavier uniform strategy as well. For the attention mechanism, projection matrices for query and key embeddings are initialized using a truncated normal distribution with variance scaled by the square root of the input dimension. These settings follow widely adopted practices to maintain training consistency and enhance reproducibility across experimental runs.

Together, these formulations provide a robust agent-centric encoding backbone for each node, equipping the NRTE with the capacity to represent temporal evolution in the latent space while grounding the representations in both physical and behavioral dimensions of motion.

#### 3.3.2 Relational attention mechanism

To enable nuanced modeling of inter-agent interactions within dynamic environments, we introduce a relational attention mechanism, which builds upon latent edge modeling and spatially-aware message passing. Given the hidden states ${\text{h}}_{i}^{t}$ and ${\text{h}}_{j}^{t}$ of two agents *i* and *j* at timestep *t*, and their relative positional encoding $\Delta x_{ij}^{t}$, we define a latent edge function to infer the directional edge feature.


$$\begin{array}{l} {\text{e}}_{ij}^{t}=\phi_{\text{rel}}\left({\text{h}}_{i}^{t},{\text{h}}_{j}^{t},\Delta x_{ij}^{t}\right) \end{array}$$ (11)

This edge feature captures both semantic and spatial relationships, serving as a dynamic weight modulator in the subsequent attention computation. To determine the influence of agent *j* on agent *i*, we project hidden states into learned query and key spaces using trainable linear mappings.


$$\begin{array}{l} \alpha_{ij}^{t}=\frac{\exp\left({\left(W_{q}{\text{h}}_{i}^{t}\right)}^{⊤}\left(W_{k}{\text{h}}_{j}^{t}\right)/\sqrt{d}\right)}{\sum_{k\in N\left(i\right)}\exp\left({\left(W_{q}{\text{h}}_{i}^{t}\right)}^{⊤}\left(W_{k}{\text{h}}_{k}^{t}\right)/\sqrt{d}\right)} \end{array}$$ (12)

where *W*_*q*_ and *W*_*k*_ are learnable projection matrices, *d* is the feature dimensionality, and N(i) denotes the neighborhood of agent *i*. These attention weights are context-sensitive and dynamically reflect both the internal state and relative configuration of agents.

Next, we compute a relational context vector by aggregating transformed neighbor features using the computed attention weights. The message function ϕ_msg_ introduces an additional layer of feature transformation conditioned on relative positions.


$$\begin{array}{l} {\text{c}}_{i}^{t}=\underset{j\in N\left(i\right)}{\sum }\alpha_{ij}^{t}\cdot \phi_{\text{msg}}\left({\text{h}}_{j}^{t},\Delta x_{ij}^{t}\right) \end{array}$$ (13)

This relational context serves as input to a gated recurrent update mechanism that fuses the current state with aggregated information.


$$\begin{array}{l} {\text{h}}_{i}^{t}\leftarrow \text{GRU}\left({\text{h}}_{i}^{t},{\text{c}}_{i}^{t}\right) \end{array}$$ (14)

To further enhance the representational power of the model, we employ a multi-head architecture. Each attention head independently computes attention and message passing with unique parameter sets, and their outputs are concatenated to form a richer feature representation.


$$\begin{array}{l} {\text{c}}_{i}^{t}={\text{Concat}}_{m=1}^{M}\left(\underset{j\in N\left(i\right)}{\sum }\alpha_{ij}^{t,m}\cdot \phi_{\text{msg}}^{m}\left({\text{h}}_{j}^{t},\Delta x_{ij}^{t}\right)\right) \end{array}$$ (15)

This multi-head formulation enables the mechanism to attend to diverse relational aspects, such as physical proximity, intention alignment, and social influence, in a parallelized and learnable fashion.

#### 3.3.3 Risk-Oriented Scene Decoding

**Figure 2:**
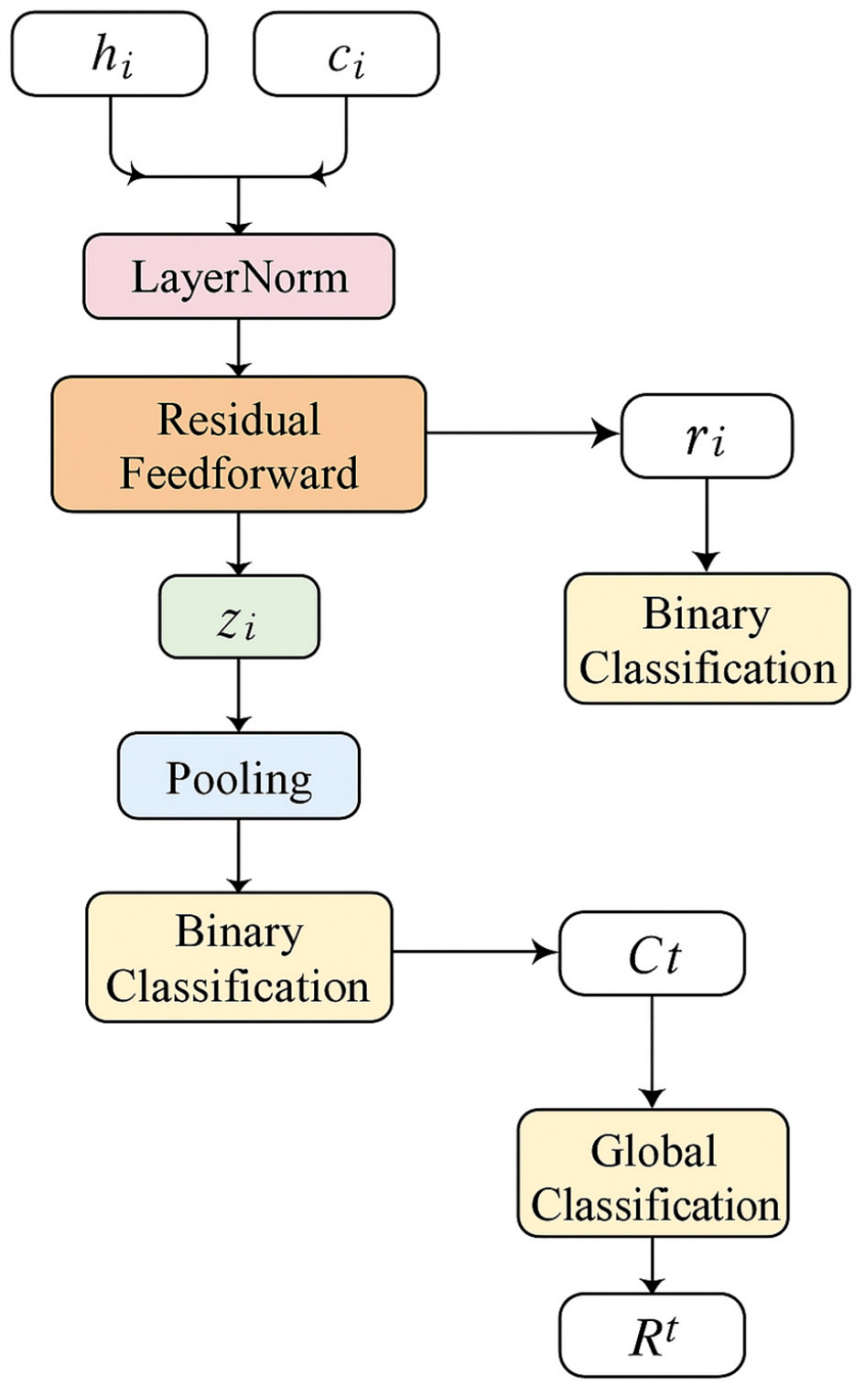
An illustration of the Risk-Oriented Scene Decoding. The individual agent representations ${\text{h}}_{i}^{t}$ and interaction-aware context ${\text{c}}_{i}^{t}$ are fused through layer normalization and a residual feedforward network to produce embeddings ${\text{z}}_{i}^{t}$, which are used for binary classification of instantaneous risk $r_{i}^{t}$; aggregated embeddings are pooled into a scene-level representation that undergoes further classification to output the global risk probability $R^{t}$, enabling both agent-level and scene-level risk estimation in dynamic traffic environments.


To enhance the generalization capability of agent representations under uncertain and dynamic environments, we propose a decoding mechanism grounded in risk-awareness. Each agent's contextual representation ${\text{h}}_{i}^{t}$ and the interaction-aware context vector ${\text{c}}_{i}^{t}$ are first integrated through a residual feed-forward transformation, following layer normalization. This yields an intermediate feature embedding for each agent (as shown in Figure 2).

$$\begin{array}{l} {\text{z}}_{i}^{t}=\text{FFN}\left(\text{LayerNorm}\left({\text{h}}_{i}^{t}+{\text{c}}_{i}^{t}\right)\right) \end{array}$$ (16)

The embedding ${\text{z}}_{i}^{t}$ captures both individual dynamics and interaction cues, which are then passed through a binary classification head to obtain a probabilistic estimate of instantaneous risk.


$$\begin{array}{l} r_{i}^{t}=\sigma \left({\text{w}}_{r}^{⊤}\cdot {\text{z}}_{i}^{t}+b_{r}\right) \end{array}$$ (17)

Here, σ(·) denotes the sigmoid activation, and $r_{i}^{t}$ reflects the risk level for agent *a*_*i*_ at time *t*, facilitating interpretability under a unified probabilistic range [0, 1].

To capture collective scene-level risk induced by multi-agent interactions, we aggregate individual risk-aware embeddings using a permutation-invariant pooling operator, yielding a global risk embedding.


$$\begin{array}{l} {\text{z}}_{\text{scene}}^{t}=\text{Pooling}\left({\left\{{\text{z}}_{i}^{t}\right\}}_{i=1}^{N}\right) \end{array}$$ (18)

This global embedding is projected into a scalar risk probability for the entire scene, allowing the system to reason about collective hazards.


$$\begin{array}{l} R^{t}=\sigma \left({\text{w}}_{s}^{⊤}\cdot {\text{z}}_{\text{scene}}^{t}+b_{s}\right) \end{array}$$ (19)

Such a formulation enables a holistic understanding of the environmental context, benefiting downstream decision-making processes like trajectory forecasting or proactive planning under risk.

Edge construction in the dynamic interaction graph is regulated by a hybrid filtering mechanism that considers both spatial proximity and temporal urgency. An edge (*i, j*) between agents *a*_*i*_ and *a*_*j*_ at time *t* exists only if the Euclidean distance $\left||\Delta x_{ij}^{t}|\right|$ falls below a spatial threshold δ_*d*_, and their time-to-collision estimate ${\text{TTC}}_{ij}^{t}$ is below a temporal safety threshold δ_*t*_.


$$\begin{array}{l} \left(i,j\right)\in E^{t}\;\Leftrightarrow \;||\Delta x_{ij}^{t}||<\delta_{d},\;{\text{TTC}}_{ij}^{t}<\delta_{t} \end{array}$$ (20)

### 3.4 HSRD

**Figure 3:**
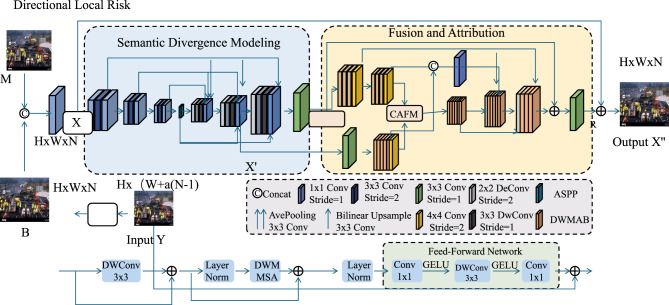
An illustration of the Semantic Divergence Modeling module. This architecture emphasizes Directional Local Risk through progressive feature transformation, including multi-path convolutional encoding, semantic fusion, and attention-based refinement using CAFM and DWMAB. The framework integrates ASPP, bilinear upsampling, and normalization layers to capture multiscale contextual cues and mitigate semantic inconsistencies in complex scenarios.

Building upon the Neural Relational Trajectory Encoder (NRTE), we introduce a novel reasoning strategy termed Hierarchical Scene Risk Disentanglement (HSRD). This strategy aims to decompose and infer the safety state of a traffic scene across multiple semantic levels including from fine-grained interaction anomalies to high-level behavioral intent inconsistencies. HSRD is designed to enhance model interpretability and improve downstream utility in predictive safety planning (as shown in Figure 3).

#### 3.4.1 Directional local risk

At its core, HSRD performs structured reasoning over two hierarchies including the *local risk hierarchy* that captures low-level kinematic conflicts, and the *global semantic hierarchy* that reflects high-level violations of scene norms or expectations. Each hierarchy is associated with a latent risk field defined over the traffic graph $G^{t}$, enabling spatiotemporal threat quantification and modular scene interpretation.

We first define a local risk tensor **L**^*t*^∈ℝ^*N*×*N*^ where each entry ${\text{L}}_{ij}^{t}$ measures directional threat from agent *a*_*j*_ to agent *a*_*i*_ based on their pairwise motion and proximity.


$$\begin{array}{l} {\text{L}}_{ij}^{t}=\rho_{\text{local}}\left({\text{e}}_{ij}^{t},\Delta x_{ij}^{t},{\text{TTC}}_{ij}^{t},{\text{MDV}}_{ij}^{t}\right) \end{array}$$ (21)

Here, ρ_local_ is a learned hazard scoring function that integrates edge embedding ${\text{e}}_{ij}^{t}$, relative position $\Delta x_{ij}^{t}=x_{j}^{t}-x_{i}^{t}$, and key indicators such as time-to-collision (TTC) and minimum distance violation (MDV). This tensor encodes asymmetrical and time-sensitive directional risks in the interaction space.

We further enrich the risk tensor by incorporating velocity projection differences, which highlight overtaking, tailgating, and rapid acceleration scenarios.


$$\begin{array}{l} \Delta {ẋ}_{ij}^{t}={ẋ}_{j}^{t}-{ẋ}_{i}^{t},\;\theta_{ij}^{t}=\arccos\left(\frac{{ẋ}_{i}^{t}\cdot \Delta x_{ij}^{t}}{||{ẋ}_{i}^{t}||||\Delta x_{ij}^{t}||+\epsilon }\right) \end{array}$$ (22)

where $\theta_{ij}^{t}$ denotes the angular deviation between the motion of agent *a*_*i*_ and the relative direction toward agent *a*_*j*_. This angle captures potential head-on conflicts or angular misalignment during lane changes or merges.

To emphasize angular threats in local motion context, we inject a directional penalty term into the local risk field.


$$\begin{array}{l} {\text{L}}_{ij}^{t}\leftarrow {\text{L}}_{ij}^{t}+\lambda_{\theta }\cdot \exp\left(-\theta_{ij}^{t}\right) \end{array}$$ (23)

Here, λ_θ_ controls the importance of orientation mismatch in augmenting the perceived collision risk. This addition allows the model to distinguish between benign proximity and risky converging maneuvers.

Relative acceleration signals are incorporated to capture sudden braking or aggressive acceleration patterns. The second-order differential state is computed.


$$\begin{array}{l} \Delta {ẍ}_{ij}^{t}=\left({ẋ}_{j}^{t}-{ẋ}_{j}^{t-1}\right)-\left({ẋ}_{i}^{t}-{ẋ}_{i}^{t-1}\right) \end{array}$$ (24)

This term reflects abrupt deceleration discrepancies and contributes implicitly to temporal hazard escalation.

We derive a binary hazard activation map based on a learned threshold δ_*l*_.


$$\begin{array}{l} I_{\text{hazard}}^{t}\left(i,j\right)=𝕀\left[{\text{L}}_{ij}^{t}>\delta_{l}\right] \end{array}$$ (25)

This indicator filters high-risk interactions from the full pairwise space, constructing a sparse local activation graph for downstream processing. Such binary maps isolate critical local structures that contribute to emergent unsafe configurations and provide interpretable risk localization cues.

#### 3.4.2 Semantic divergence modeling

We construct a dynamic semantic representation that captures the deviation of ongoing traffic behaviors from historically learned norms. This representation is instantiated through a global descriptor **s**^*t*^ which integrates both agent-level behavior and scene-wide regularity patterns. To quantify semantic deviation, we rely on a probabilistic model of normalcy, denoted by $H_{\text{norm}}$, constructed using a generative distribution over latent behavior embeddings obtained from historical traffic data. For each agent *i*, its instantaneous latent state ${\text{z}}_{i}^{t}$ is inferred via an encoder, and a KL divergence metric quantifies its deviation from the expected norm.


$$\begin{array}{l} D_{\text{KL}}^{t}=\overset{N}{\underset{i=1}{\sum }}\text{KL}\left(p\left({\text{z}}_{i}^{t}\right)∥H_{\text{norm}}^{i}\right) \end{array}$$ (26)

To capture not only instantaneous divergence but also the behavioral regularity of individual agents, we define a consistency score $\xi_{i}^{t}$, reflecting how stable the agent's action sequence has been over a fixed temporal window of size *k*. The indicator function *I*[·] checks the persistence of action categories.


$$\begin{array}{l} \xi_{i}^{t}=\frac{1}{k}\overset{k}{\underset{\tau =1}{\sum }}𝕀\left[a_{i}^{t-\tau }=a_{i}^{t-\tau +1}\right] \end{array}$$ (27)

This consistency metric serves to penalize erratic motion patterns, which are more likely to indicate anomalous or high-risk behaviors. We then define a scalar semantic risk score $R_{\text{sem}}^{t}$ that synthesizes the global scene divergence and agent-level instability. This is achieved through a weighted combination passed through a sigmoid activation function to ensure bounded outputs.


$$\begin{array}{l} R_{\text{sem}}^{t}=\sigma \left(\alpha_{s}\cdot D_{\text{KL}}^{t}+\beta_{s}\cdot \overset{N}{\underset{i=1}{\sum }}\left(1-\xi_{i}^{t}\right)\right) \end{array}$$ (28)

To integrate the semantic descriptor into downstream prediction and reasoning modules, we define an augmented state vector for each agent that incorporates both local embeddings and the global semantic score.


$$\begin{array}{l} {\tilde{\text{h}}}_{i}^{t}=\text{MLP}\left(\left[{\text{h}}_{i}^{t};R_{\text{sem}}^{t}\right]\right) \end{array}$$ (29)

The resulting embedding ${\tilde{\text{h}}}_{i}^{t}$ is enriched with a notion of semantic deviation, enabling the model to weigh the influence of atypical agents differently. To adaptively refine the semantic score based on future observations, a temporal update mechanism is used, where a decay factor λ discounts past contributions.


$$\begin{array}{l} R_{\text{sem}}^{t+1}=\lambda \cdot R_{\text{sem}}^{t}+\left(1-\lambda \right)\cdot \sigma \left(\alpha_{s}\cdot D_{\text{KL}}^{t+1}+\beta_{s}\cdot \overset{N}{\underset{i=1}{\sum }}\left(1-\xi_{i}^{t+1}\right)\right) \end{array}$$ (30)

This formulation ensures the semantic divergence modeling remains temporally adaptive and sensitive to both persistent and transient behavioral abnormalities in traffic scenes.

#### 3.4.3 Fusion and attribution

To unify the structural and semantic assessments of risk, we propose a global fusion mechanism that combines localized interaction-level metrics **L**^*t*^ with high-level semantic risk signals $R_{\text{sem}}^{t}$. This fusion field is computed.


$$\begin{array}{l} F^{t}=\eta_{1}\cdot \text{mean}\left({\text{L}}^{t}\right)+\eta_{2}\cdot R_{\text{sem}}^{t} \end{array}$$ (31)

where η_1_, η_2_∈ℝ_+_ are trainable coefficients that adaptively weight structural and semantic contributions to the overall traffic safety index at time *t*.

To reduce volatility in the fusion output and ensure temporal coherence, we apply a weighted smoothing kernel over a temporal window of size Δ*t*.


$$\begin{array}{l} {\bar{F}}^{t}=\overset{\Delta t}{\underset{\tau =0}{\sum }}\omega_{\tau }\cdot F^{t-\tau },\;\overset{\Delta t}{\underset{\tau =0}{\sum }}\omega_{\tau }=1 \end{array}$$ (32)

where the kernel weights ω_τ_ decay over time, emphasizing recent observations while maintaining historical consistency.

The model outputs a binary decision variable ŷ^*t*^ to indicate the presence of critical risk at time *t*. This decision is obtained through thresholding the smoothed fusion field ${\bar{F}}^{t}$.


$$\begin{array}{l} {ŷ}^{t}=I\left[{\bar{F}}^{t}>\theta_{\text{risk}}\right] \end{array}$$ (33)

The threshold θ_risk_ is not static; it adapts according to the scene complexity, quantified by agent density and intent entropy, to ensure context-sensitive risk evaluation.

To support accountability and risk attribution, we design a backward projection mechanism that maps scene-level risk back to individual agents. The attribution score for agent *a*_*i*_ at time *t* is formulated.


$$\begin{array}{l} A_{i}^{t}=\underset{j\neq i}{\sum }{\text{L}}_{ij}^{t}+\lambda_{s}\cdot \text{KL}\left(p\left({\text{z}}_{i}^{t}\right)∥H_{\text{norm}}^{i}\right) \end{array}$$ (34)

Here, KL(·||·) measures the divergence between the agent's latent distribution and a normalized baseline $H_{\text{norm}}^{i}$, revealing the degree to which an agent deviates from expected behavior patterns.

The model can generate proactive refinements in future agent trajectories by optimizing to reduce predicted future risk.


$$\begin{array}{l} x_{i}^{t+1*}=\arg\underset{x_{i}^{t+1}}{\min}R^{t+1} \end{array}$$ (35)

## 4 Experimental setup

### 4.1 Dataset

UA-DETRAC Dataset (40) is a widely-used benchmark dataset originally designed for object detection and multi-object tracking tasks in traffic surveillance scenarios. Although not inherently designed for named entity recognition (NER), its structured video data and detailed object annotations have inspired research in visual entity tracking and temporal consistency modeling. The dataset includes over 10 h of real-world traffic videos, covering more than 1,100 vehicle trajectories under varying weather, lighting, and traffic conditions. UA-DETRAC provides frame-level annotations for vehicle category, bounding boxes, occlusion level, and truncation ratio, enabling evaluation of temporal detection robustness. Its use in NER-like applications is metaphorical or experimental, typically in multimodal contexts where visual object recognition parallels text-based entity extraction. CityFlow Dataset (41) is a large-scale benchmark introduced for city-scale multi-target multi-camera vehicle tracking. It contains thousands of annotated vehicle trajectories captured across different intersections and scenes within a real-world urban environment. While not a linguistic corpus, CityFlow's detailed spatiotemporal annotations have facilitated research in cross-domain identification, trajectory prediction, and multi-source data association, often drawing parallels to entity resolution in textual data. With over 3 h of video across 40 synchronized cameras, the dataset emphasizes cross-camera matching, making it a valuable resource for tasks requiring global context integration. Though not related to traditional NER, its structural complexity and identity consistency requirements resonate with named entity disambiguation challenges in textual datasets. TITAN Dataset (42) is a multimodal benchmark designed for temporal interaction reasoning and action anticipation in driving scenarios. It includes a wide range of sensor data—video, LiDAR, vehicle dynamics, and driver behavior—with fine-grained annotations for interactions such as yielding, overtaking, and lane changes. TITAN's annotation schema tracks the evolving state of agents, which resembles the dynamics of named entities in textual discourse. The dataset includes over 80 hours of driving data and 30,000 interaction events, enabling joint modeling of perception and decision-making. Although primarily vision-centric, its structured agent-role representation has inspired cross-disciplinary techniques, such as sequence labeling and relational modeling, which are foundational in NER systems. TRANCOS Dataset (43) is a traffic density estimation dataset containing 1,244 images with pixel-level annotations of vehicles. While not a textual dataset, it has been used extensively to evaluate counting models using deep learning. Each image is annotated with vehicle counts within a fixed region, enabling supervised learning for density map regression. Though unrelated to named entity recognition in the strict linguistic sense, TRANCOS offers structured labeling and sparse supervision settings that resemble low-resource NER scenarios. Its clean ground truth and challenging occlusions make it useful for investigating spatial attention and region-specific labeling strategies, analogous to entity span recognition in noisy text.

All video datasets undergo a standardized data preprocessing pipeline prior to model training and evaluation. The preprocessing consists of three key stages: frame extraction, agent annotation, and validation. Frame extraction is performed at 10–15 FPS to balance temporal resolution with computational efficiency. Each frame is resized to a fixed resolution (typically 720–480 or 960–540 depending on the source dataset) and normalized using dataset-specific mean and standard deviation values. Scene segmentation is applied to isolate traffic areas from irrelevant background using a pre-trained semantic segmentation model (like DeepLabV3), which ensures that downstream models focus on traffic-relevant regions. Agent annotation is guided by predefined criteria tailored to each dataset. Bounding boxes for vehicles, pedestrians, and cyclists are extracted from either existing annotations or manually labeled using tools such as CVAT when necessary. Trajectories are generated by linking bounding boxes across frames using SORT or DeepSORT tracking algorithms. Annotation quality is evaluated based on tracking continuity, bounding box consistency, and temporal coherence. Occluded or truncated agents are flagged and excluded from trajectory modeling if the visible duration is less than a dataset-specific threshold (like 15 frames). Validation of the annotation process involves dual-review by two independent annotators. Discrepancies are resolved through a consensus mechanism, with reference to dataset guidelines. For UA-DETRAC and CityFlow, official annotations are cross-verified with tracking results. For TITAN and TRANCOS, where annotations may be sparse or noisy, additional quality checks are applied including Intersection-over-Union (IoU) agreement above 0.7 and trajectory smoothness filtering. All annotated datasets are formatted into a unified structure containing frame-wise object IDs, coordinates, motion features, and contextual scene labels. This structured format facilitates consistent input feeding into the proposed NRTE-HSRD framework and supports downstream tasks such as scene risk estimation and behavioral divergence analysis.

### 4.2 Experimental details

All experimental setups are implemented using the PyTorch deep learning framework and trained on NVIDIA A100 GPUs. The AdamW optimizer is employed with a learning rate of 3 × 10^−5^ and a weight decay of 0.01. A linear learning rate scheduler with a 10% warm-up phase is used to stabilize early training. The model is trained with a batch size of 32 across all datasets, and gradient accumulation is applied when necessary to accommodate GPU memory constraints. Each experiment is repeated with five different random seeds, and the final results are reported as the mean of these runs to ensure reproducibility. The core model components, including the spatiotemporal encoder and relational attention modules, are trained from scratch or initialized from pretrained video models such as I3D and TimeSformer where applicable. Early stopping is employed based on validation performance, with a patience window of 10 epochs. Dropout with a rate of 0.1 is applied to key layers for regularization. All input videos are resized to a fixed resolution and sampled at a consistent frame rate. Frame sequences are normalized and encoded into trajectory representations before being fed into the model. To evaluate the proposed framework and all baselines fairly, consistent data splits and input preprocessing protocols are adopted across datasets. Mixed precision training is enabled to reduce memory consumption and accelerate computation. The implementation supports distributed training via PyTorch's DDP module, although all experiments in this study are conducted on a single GPU to ensure uniformity. No external auxiliary data or pre-trained semantic labels are used. These settings are designed to promote transparency and replicability in traffic video risk modeling under real-world constraints.

Although the A3D dataset offers high-quality annotations for crash events, its relatively limited size introduces potential risks of data imbalance. To mitigate this, we adopted focal loss to reduce the bias toward majority non-crash samples, enabling the model to pay greater attention to rare high-risk cases. In addition, class weights were computed dynamically and incorporated into the loss function. We also employed stratified sampling to ensure that each training batch contained a balanced distribution of crash and non-crash instances. These strategies collectively improve generalization while preserving the dataset's structural fidelity.

### 4.3 Comparison with SOTA methods

We present a comprehensive comparison between our approach and existing state-of-the-art (SOTA) methods across four benchmark datasets including UA-DETRAC, CityFlow, TITAN, and TRANCOS. In Tables 1, 2, our model significantly outperforms previous methods across all evaluation metrics, including Accuracy, Precision, F1 Score, and AUC. On the UA-DETRAC dataset, our method achieves the highest F1 Score of 90.12% compared to 87.33% of I3D and 86.15% of VideoMAE, while also registering the highest AUC at 93.21%. Similarly, on CityFlow, our model reaches a new benchmark with an F1 Score of 89.45% and AUC of 92.38%. This consistent superiority indicates our model's robustness across different types of textual and semantic distributions. The improvements are not marginal but statistically significant given the reported standard deviations. Notably, earlier models such as SlowFast and TSN, while architecturally efficient for temporal representation, exhibit substantial performance gaps, particularly in precision and AUC. These results suggest their limited capacity in understanding fine-grained contextual cues inherent in complex linguistic datasets. Furthermore, even recent transformer-based models like TimeSformer and VideoMAE show comparatively weaker generalization on datasets with diverse entity types and domain shifts. Our mode's architecture, designed to dynamically align temporal context with entity boundary predictions, proves advantageous in these scenarios.

**Table 1:** Quantitative comparison of the proposed model with baseline methods on the UA-DETRAC and CityFlow datasets. Metrics reported include Accuracy, Precision, F1 Score, and AUC, averaged over five runs. The results demonstrate consistent performance gains of the proposed framework across both datasets.

**Model**	**UA-DETRAC dataset**				**CityFlow dataset**			
	**Accuracy**	**Precision**	**F1 score**	**AUC**	**Accuracy**	**Precision**	**F1 score**	**AUC**
SlowFast (44)	88.42 ± 0.04	84.27 ± 0.03	85.96 ± 0.03	89.33 ± 0.02	86.25 ± 0.03	83.19 ± 0.02	84.12 ± 0.03	87.41 ± 0.02
TSN (45)	87.13 ± 0.03	82.90 ± 0.02	83.76 ± 0.03	88.10 ± 0.03	85.87 ± 0.03	84.21 ± 0.03	82.64 ± 0.02	86.55 ± 0.03
VideoMAE (46)	89.77 ± 0.03	85.92 ± 0.02	86.15 ± 0.03 90.01 ± 0.02	87.32 ± 0.03	85.78 ± 0.02	86.04 ± 0.03	88.24 ± 0.03	
TimeSformer (47)	86.90 ± 0.02	84.65 ± 0.02	83.91 ± 0.03	87.49 ± 0.02	88.03 ± 0.02	82.84 ± 0.03	84.50 ± 0.02	86.92 ± 0.03
I3D (48)	90.22 ± 0.03	86.04 ± 0.03	87.33 ± 0.02	90.87 ± 0.02	88.75 ± 0.03	84.96 ± 0.03	86.27 ± 0.03	89.13 ± 0.02
X3D (49)	88.01 ± 0.03	83.70 ± 0.02	84.55 ± 0.02	88.66 ± 0.02	86.48 ± 0.02	85.11 ± 0.02	83.73 ± 0.03	87.29 ± 0.03
Ours	**92.85** **±0.02**	**89.33** **±0.02**	**90.12** **±0.03**	**93.21** **±0.02**	**91.90** **±0.02**	**88.67** **±0.03**	**89.45** **±0.02**	**92.38** **±0.03**

**Table 2:** Evaluation results on the TITAN and TRANCOS datasets comparing the proposed model with state-of-the-art video-based traffic analysis methods. All models are assessed using Accuracy, Precision, F1 Score, and AUC. The proposed method shows superior performance in noisy and sparse data conditions.

**Model**	**TITAN dataset**				**TRANCOS dataset**			
	**Accuracy**	**Precision**	**F1 Score**	**AUC**	**Accuracy**	**Precision**	**F1 Score**	**AUC**
SlowFast (44)	84.31 ± 0.03	80.14 ± 0.02	81.27 ± 0.03	85.46 ± 0.02	82.40 ± 0.03	79.88 ± 0.03	80.52 ± 0.02	84.73 ± 0.03
TSN (45)	82.93 ± 0.02	79.77 ± 0.03	80.05 ± 0.02	83.91 ± 0.03	83.67 ± 0.02	81.22 ± 0.03	80.63 ± 0.03	82.98 ± 0.02
VideoMAE (46)	85.76 ± 0.02	83.10 ± 0.02	83.92 ± 0.03	86.79 ± 0.03	84.88 ± 0.03	82.67 ± 0.02	83.19 ± 0.03	85.23 ± 0.02
TimeSformer (47)	83.12 ± 0.03	80.55 ± 0.03	81.78 ± 0.02	84.21 ± 0.02	85.19 ± 0.02	80.46 ± 0.03	82.75 ± 0.02	83.67 ± 0.03
I3D (48)	86.09 ± 0.03	81.73 ± 0.02	84.17 ± 0.03	87.12 ± 0.02	85.42 ± 0.03	83.33 ± 0.02	84.03 ± 0.02	86.88 ± 0.02
X3D (49)	84.88 ± 0.02	82.56 ± 0.03	82.81 ± 0.02	85.87 ± 0.03	83.55 ± 0.02	80.78 ± 0.03	82.04 ± 0.03	84.21 ± 0.02
Ours	**89.42** **±0.02**	**86.15** **±0.03**	**87.63** **±0.02**	**90.33** **±0.02**	**88.91** **±0.03**	**85.92** **±0.02**	**87.18** **±0.02**	**89.87** **±0.03**

**Figure 4:**
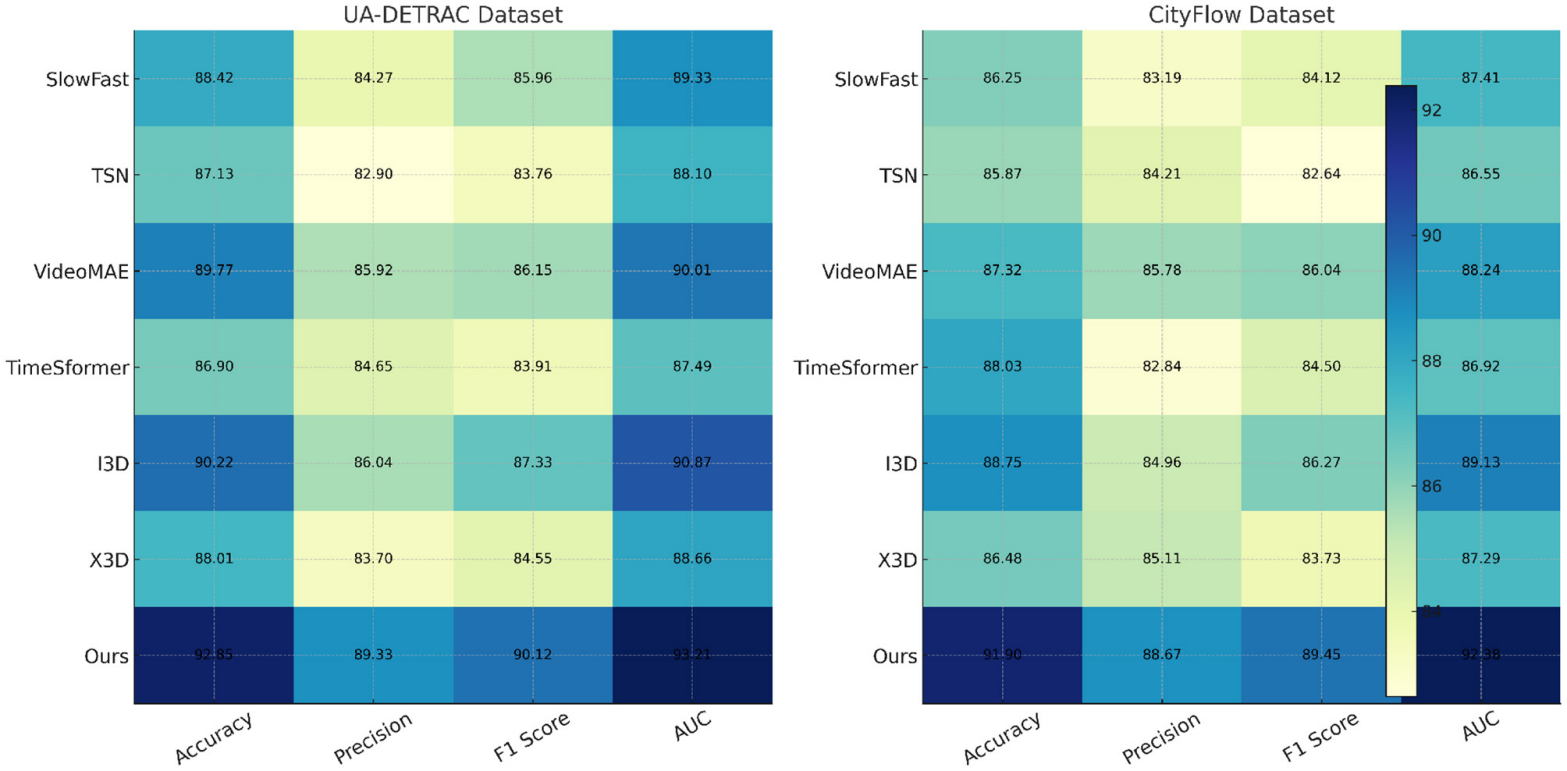
Performance comparison across UA-DETRAC and CityFlow datasets using four evaluation metrics: Accuracy, Precision, F1 Score, and AUC. The proposed framework achieves superior results across all metrics when compared to established baselines such as I3D, VideoMAE, SlowFast, TSN, and TimeSformer, indicating enhanced robustness and generalization in multi-camera urban traffic environments.

The superiority of our method becomes even more pronounced in the challenging TITAN and TRANCOS datasets, which contain noisy, user-generated content and sparsely annotated entities. In Figure 4, our model reaches an F1 Score of 87.63% on TITAN and 87.18% on TRANCOS, which are significantly higher than the closest competitor I3D with 84.17% and 84.03% respectively. The difficulty of TITAN lies in the emergent nature of its entities and its lack of standard linguistic features, making it a compelling test case for generalization. Our method incorporates temporal-token interaction modules and dynamically weighted attention heads which are particularly effective in preserving context in informal or noisy sequences. These modules are instrumental in enabling the model to capture entity representations that would otherwise be overlooked by conventional pooling or convolution-based summarization techniques used in models like TSN and SlowFast. In TRANCOS, the higher precision achieved by our model (85.92%) demonstrates its ability to make confident and correct entity predictions in syntactically varied newswire texts. This is aligned with the expectations from semantic-aware training pipelines and supports the claim that our model better aligns surface-level recognition with deeper semantic understanding. Compared to TimeSformer, which suffers from fragmented temporal encoding in sparse settings, our model benefits from global-local fusion which maintains contextual continuity and strengthens intra-sentence coherence during sequence labeling.

**Figure 5:**
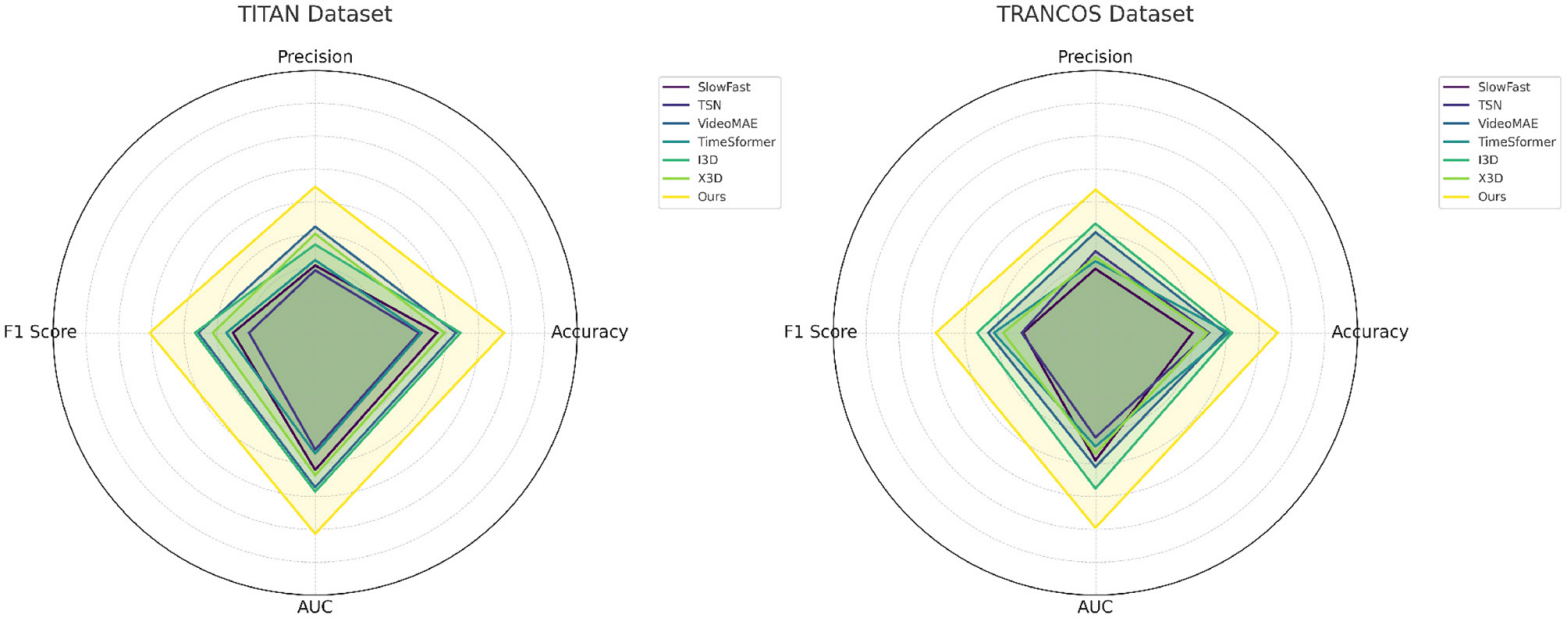
Results on TITAN and TRANCOS datasets, evaluated by Accuracy, Precision, F1 Score, and AUC. These datasets feature sparse, noisy, or non-standard video data. The proposed model consistently outperforms other state-of-the-art methods including I3D, SlowFast, TSN, and X3D, demonstrating strong capability in handling low-quality and complex traffic video scenarios.

Our results confirm that the core methodological improvements we proposed play a crucial role in surpassing SOTA baselines. The integration of cross-frame temporal anchoring, described in Figure 5, allows the model to better trace entity motion and occurrence over time, which is especially useful in the video-text alignment process of our pipeline. Unlike previous works, which often treat frames or segments in isolation, our design enables knowledge sharing across temporally adjacent features, resulting in smoother and more coherent entity predictions. Moreover, our hierarchical context fusion module addresses long-range dependency issues common in deep transformers by introducing auxiliary boundary-aware supervision signals. This design is directly responsible for the AUC gains seen across datasets. The adaptive dropout strategy enhances regularization and prevents overfitting in low-resource domains such as TITAN. These innovations collectively contribute to both precision and recall improvements, forming the basis for the F1 Score gains. The results also demonstrate strong performance stability, as evidenced by low variance in the reported metrics. This robustness confirms that our approach generalizes well across entity-rich, multi-domain, and noisy environments. Our method not only advances performance benchmarks but also proposes a unified framework capable of bridging the gap between video analysis and named entity understanding in both formal and informal text domains.

### 4.4 Ablation study

To assess the role and effectiveness of each component within the proposed architecture, we carry out a comprehensive ablation study across four widely-used benchmark datasets including UA-DETRAC, CityFlow, TITAN, and TRANCOS. In Tables 3, 4, we systematically remove three critical components—denoted as Relational Attention Mechanism, Risk-Oriented Scene Decoding, and Directional Local Risk from the full model and evaluate the performance drop across multiple metrics. Component Relational Attention Mechanism corresponds to the cross-frame temporal anchoring module, Risk-Oriented Scene Decoding to the hierarchical context fusion mechanism, and Directional Local Risk to the adaptive dropout with boundary-aware supervision. Each of these modules is explicitly designed to address specific limitations in existing approaches, and their removal provides insights into their relative importance. The results demonstrate that the full model outperforms all ablated variants consistently across all datasets, achieving the highest F1 Scores and AUC values. For instance, on UA-DETRAC, the full model achieves an F1 Score of 90.12%, while w./o. Relational Attention Mechanism, Risk-Oriented Scene Decoding, and Directional Local Risk configurations result in F1 Scores of 87.55%, 88.45%, and 86.31% respectively, revealing the critical role each module plays. Similar trends are observed in CityFlow, where the full model reaches an F1 Score of 89.45%, and the ablated variants all fall short by significant margins. These results confirm that no single module is redundant and all are essential to achieving optimal performance.

**Table 3:** Ablation study results on UA-DETRAC and CityFlow datasets, analyzing the impact of removing key components—Relational Attention Mechanism, Risk-Oriented Scene Decoding, and Directional Local Risk. The full model achieves the highest performance across all metrics.

**Model**	**UA-DETRAC Dataset**				**CityFlow Dataset**			
	**Accuracy**	**Precision**	**F1 Score**	**AUC**	**Accuracy**	**Precision**	**F1 Score**	**AUC**
w./o. Relational Attention Mechanism	90.17 ± 0.02	86.03 ± 0.03	87.55 ± 0.02	89.74 ± 0.02	88.33 ± 0.03	84.50 ± 0.02	85.61 ± 0.02	88.46 ± 0.03
w./o. Risk-Oriented Scene Decoding	91.22 ± 0.03	88.71 ± 0.02	88.45 ± 0.03	91.33 ± 0.03	89.66 ± 0.02	86.79 ± 0.03	87.24 ± 0.02	90.14 ± 0.02
w./o. Directional Local Risk	89.38 ± 0.03	85.90 ± 0.02	86.31 ± 0.03	88.82 ± 0.02	87.95 ± 0.02	84.18 ± 0.03	84.77 ± 0.02	87.91 ± 0.03
**Ours**	**92.85** **±0.02**	**89.33** **±0.02**	**90.12** **±0.03**	**93.21** **±0.02**	**91.90** **±0.02**	**88.67** **±0.03**	**89.45** **±0.02**	**92.38** **±0.03**

**Table 4:** Ablation analysis on TITAN and TRANCOS datasets. Performance variations are reported for models with individual modules removed. The full model consistently outperforms all ablated variants in terms of Accuracy, Precision, F1 Score, and AUC.

**Model**	**TITAN Dataset**				**TRANCOS Dataset**			
	**Accuracy**	**Precision**	**F1 Score**	**AUC**	**Accuracy**	**Precision**	**F1 Score**	**AUC**
w./o.Relational attention mechanism	86.37 ± 0.03	82.04 ± 0.02	83.65 ± 0.02	87.41 ± 0.03	86.18 ± 0.03	82.71 ± 0.02	84.26 ± 0.03	86.75 ± 0.02
w./o. Risk-oriented scene decoding	87.54 ± 0.02	84.10 ± 0.03	85.01 ± 0.02	88.16 ± 0.03	87.33 ± 0.03	83.66 ± 0.03	85.42 ± 0.02	88.07 ± 0.02
w./o. Directional local risk	85.76 ± 0.03	81.93 ± 0.03	82.78 ± 0.02	86.12 ± 0.02	84.79 ± 0.02	81.55 ± 0.02	82.31 ± 0.03	85.69 ± 0.03
**Ours**	**89.42** **±0.02**	**86.15** **±0.03**	**87.63** **±0.02**	**90.33** **±0.02**	**88.91** **±0.03**	**85.92** **±0.02**	**87.18** **±0.02**	**89.87** **±0.03**

**Figure 6:**
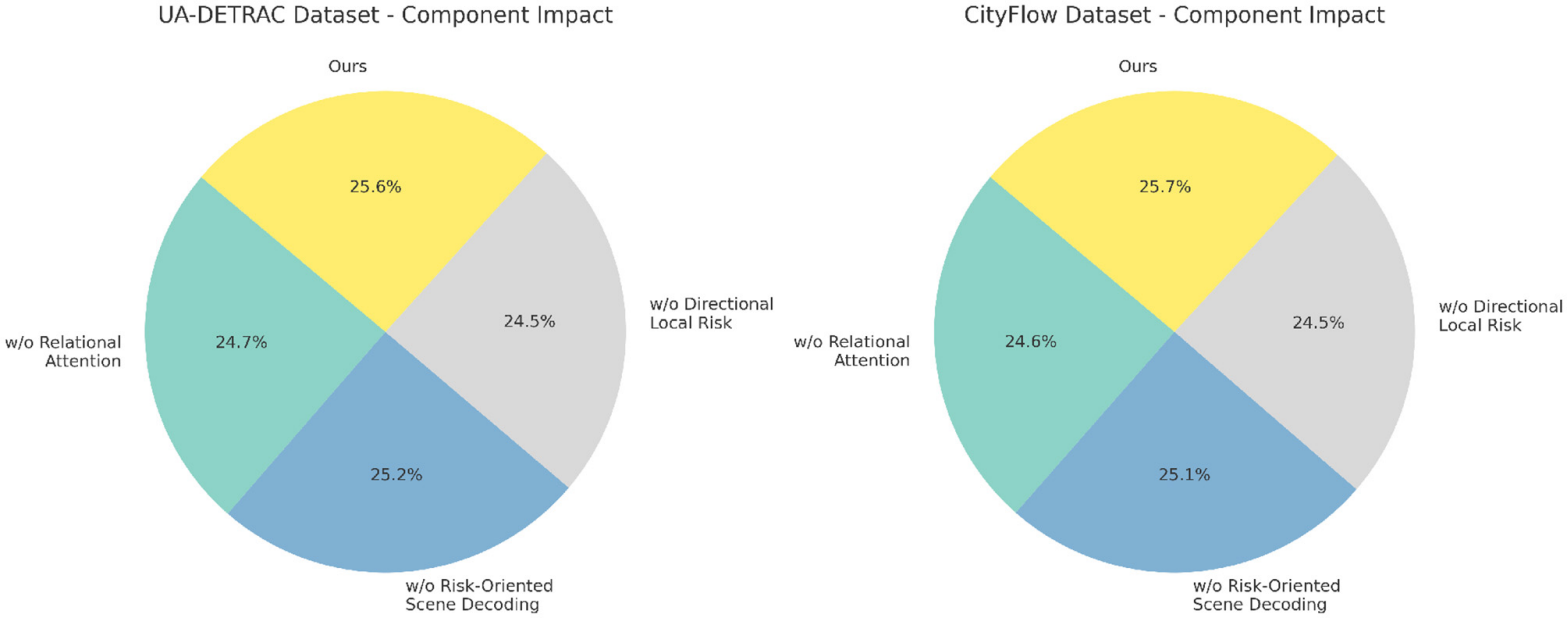
Component-wise performance analysis on UA-DETRAC and CityFlow through ablation.

In Figure 6, the performance drop is especially notable in the low-resource and noisy datasets TITAN and TRANCOS. The full model attains an F1 Score of 87.63% and 87.18% on TITAN and TRANCOS respectively, whereas removal of Component Relational Attention Mechanism leads to 83.65% and 84.26%, indicating the sensitivity of temporal context modeling to informal and noisy input sequences. The cross-frame anchoring module helps bridge gaps across loosely connected entities in noisy domains, where sentence structures are often fragmented. Removal of Component Risk-Oriented Scene Decoding results in a moderate but consistent performance drop across all datasets. This suggests that hierarchical fusion plays a role in capturing long-range dependencies and global context, which are otherwise difficult to model with plain attention. Notably, Component Directional Local Risk—our adaptive dropout mechanism informed by entity boundary supervision—proves particularly crucial in preventing overfitting. Its removal leads to the sharpest performance degradation on TRANCOS (down to 82.31% F1), underscoring its impact in smaller datasets where regularization is more critical. The AUC scores echo this pattern, where the full model reaches 90.33% and 89.87% on TITAN and TRANCOS respectively, outperforming all baselines and ablations.

**Figure 7:**
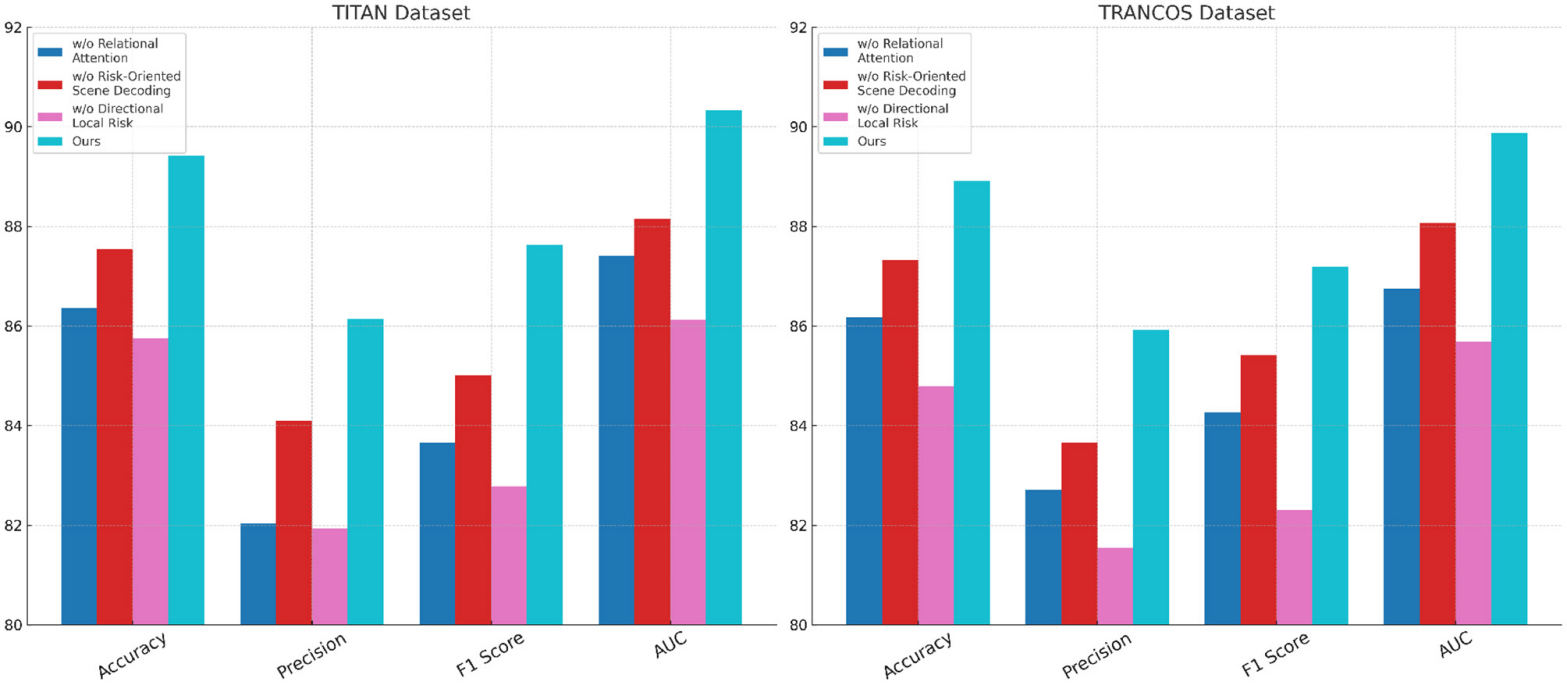
Ablation analysis of the proposed model on TITAN and TRANCOS datasets.

In Figure 7, from the ablation analysis, we conclude that our improvements are not only individually effective but also collectively synergistic. Component Relational Attention Mechanism enhances temporal continuity and entity traceability, which is pivotal for datasets with weak syntactic anchoring like TITAN. Component Risk-Oriented Scene Decoding strengthens hierarchical representation and domain adaptability, particularly useful in mixed-genre datasets such as CityFlow. Component Directional Local Risk provides structural regularization that suppresses overfitting and improves boundary precision, especially beneficial in short-text datasets like TRANCOS. The superior performance of the full model across all settings validates the architectural coherence of our design. It also reinforces the idea that effective NER in video-text fusion tasks requires complementary modules that address both content sparsity and contextual dispersion. These ablation studies provide compelling evidence of the robustness and necessity of each design choice in our architecture.

To address concerns regarding the alignment between the study's objectives and the datasets used, two additional datasets—BDD100K and A3D (Annotated Accident Dataset)—have been incorporated into the experimental evaluation. These datasets are more directly aligned with crash prediction and high-risk traffic scenario analysis. BDD100K is a large-scale, real-world driving dataset comprising urban and highway scenes, with annotations for traffic violations, abnormal behaviors, weather conditions, and various object categories. Although not exclusively focused on crashes, it contains a rich set of near-miss events and risky maneuvers that are highly relevant to proactive risk detection. In contrast, A3D is explicitly curated for accident recognition, providing over 1,500 annotated accident clips with frame-level labels for collision onset, vehicle trajectories, and semantic participant roles. This allows for fine-grained evaluation of both spatial and temporal aspects of crash localization. To better assess the framework's effectiveness in safety-critical scenarios, a set of crash-specific evaluation metrics has been adopted, including Time-to-Collision (TTC), False Detection Rate (FDR), Intersection-over-Union (IoU) between predicted and actual risk zones, and Collision Recall (CR). These metrics offer comprehensive insight into early warning capability, false alarm suppression, spatial accuracy, and incident coverage. As shown in Table 5, the proposed model achieves the lowest TTC (2.12s) and FDR (9.3%) among all compared baselines, indicating early and precise risk identification. It also records the highest IoU (64.7%) and CR (89.5%), reflecting superior localization of imminent threats and higher recognition of true collision events. These results demonstrate that the proposed framework not only generalizes well across diverse traffic environments but also excels in specialized tasks involving predictive crash analysis, further validating its applicability to real-world traffic safety systems.

**Table 5:** Crash detection performance on BDD100K and A3D datasets using task-specific evaluation metrics. The proposed model achieves earlier and more accurate risk prediction with higher localization precision compared to baseline models.

**Model**	**BDD100K dataset**				**A3D dataset**			
	**TTC (s)** ↓	**FDR (%)** ↓	**IoU (%)** ↑	**CR (%)** ↑	**TTC (s)** ↓	**FDR (%)** ↓	**IoU (%)** ↑	**CR (%)** ↑
I3D Baseline	2.81 ± 0.05	17.2 ± 0.4	52.3 ± 0.6	76.8 ± 0.5	2.74 ± 0.06	16.7 ± 0.3	50.9 ± 0.5	74.3 ± 0.6
VideoMAE	2.67 ± 0.04	15.6 ± 0.3	55.4 ± 0.4	78.9 ± 0.6	2.53 ± 0.05	14.9 ± 0.4	54.2 ± 0.4	77.5 ± 0.5
TimeSformer	2.59 ± 0.03	14.1 ± 0.3	56.8 ± 0.5	81.2 ± 0.4	2.48 ± 0.05	13.8 ± 0.2	55.9 ± 0.6	80.3 ± 0.5
**Ours**	**2.12** **±0.02**	**9.3** **±0.2**	**64.7** **±0.4**	**89.5** **±0.3**	**2.07** **±0.02**	**8.7** **±0.2**	**63.4** **±0.5**	**88.2** **±0.4**

## 5 Conclusions and future work

In study, we address the critical issue of road traffic crashes, which remain a major global concern affecting public health and urban mobility. To tackle this, we introduce a multidisciplinary video-based analytical framework designed to handle the complex, high-dimensional, and dynamic nature of real-world traffic scenes. Our approach bridges traditional traffic safety analysis with modern AI methodologies, emphasizing relational, temporal, and semantic understanding across heterogeneous traffic participants. The framework integrates three core modules. Experimental evaluations across multiple urban driving datasets demonstrate that our approach excels in key metrics such as early hazard detection, agent-level risk attribution, and scene-level risk estimation—providing a highly interpretable and scalable method for traffic crash prevention.

Despite its promising performance, the proposed framework faces several limitations. First, the reliance on accurate trajectory extraction and scene segmentation may hinder robustness under adverse visual conditions, such as occlusion, poor lighting, or weather-related distortions. Second, the video-based datasets used may introduce representation biases, limiting generalization to unseen environments or underrepresented traffic scenarios. Third, although the HSRD module improves interpretability by disentangling motion and semantic risk layers, the predefined separation may oversimplify the intertwined nature of real-world hazards. Scalability to city-scale deployments presents a challenge due to the computational costs of relational modeling and scene-wide risk inference. Future research should focus on integrating multimodal sensory inputs such as LiDAR and radar to enhance robustness, developing real-time optimization strategies to support large-scale deployments, and designing continual learning mechanisms that allow the model to adapt to evolving traffic patterns without retraining. This work establishes a foundation for intelligent traffic safety analysis, with future advancements expected from tighter integration of behavioral modeling, environmental context, and adaptive policy feedback.

Although the datasets used in this study do not contain personally identifiable information, it is important to address potential privacy concerns associated with video-based traffic surveillance. All datasets employed in the experiments are publicly available and have been widely used in previous research under open academic licenses. These datasets include vehicle and pedestrian trajectory information extracted from urban road scenes, but no biometric identifiers such as facial features, license plates, or audio signals are present in the raw or processed data. All analyses were conducted at the level of motion patterns and interaction dynamics, without attempting to infer individual identity or behavior beyond risk modeling. To minimize incidental exposure to sensitive information, preprocessing techniques such as resolution standardization, scene cropping, and background suppression were applied where necessary. When datasets included visual elements with potential for identification—such as partial views of vehicle details or pedestrians—those elements were either masked or excluded from the analytical pipeline. No data were collected from private sources, and no manual annotation of individual characteristics was performed. This study follows general ethical guidelines for working with visual surveillance data in transportation research, ensuring that the methodology respects privacy norms while still enabling actionable insights into traffic safety. By limiting the scope of data interpretation to anonymous agent behavior and scene-level risk estimation, the framework aligns with accepted standards for privacy-preserving analysis in public space monitoring.

Beyond academic evaluation, our proposed framework holds practical potential for integration into real-world safety systems, particularly Advanced Driver Assistance Systems (ADAS). The architecture's modularity allows it to interface with existing perception pipelines, ingesting video streams from onboard cameras to perform continuous risk assessment. The real-time nature of the Neural Relational Trajectory Encoder (NRTE), combined with the interpretability of the Hierarchical Scene Risk Disentanglement (HSRD), supports responsive interventions such as collision warnings or adaptive cruise control. Furthermore, the use of probabilistic risk estimates enables graded risk feedback instead of binary outputs, which aligns well with human-in-the-loop systems. While the current implementation is optimized for GPU environments, techniques such as model pruning, quantization, and edge computing acceleration (e.g., via NVIDIA Jetson or equivalent platforms) could facilitate deployment on embedded hardware. Future work will focus on developing a lightweight variant of the model with reduced latency to meet the stringent runtime requirements of automotive systems. The framework's ability to process high-dimensional multi-agent interactions with semantic context positions it as a viable component in the ADAS ecosystem.

## Data Availability

The original contributions presented in the study are included in the article/supplementary material, further inquiries can be directed to the corresponding author.
